# From Concept to Classroom: Developing and Evaluating Pain Smart, a School-Based Pain Education Module

**DOI:** 10.1080/24740527.2026.2720684

**Published:** 2026-10-26

**Authors:** Isabelle Bogard, Steven J. Kamper, Andrew Sortwell, Laura R.C. Montgomery, Christopher M. Williams, Justin Costello, Tie P. Yamato

**Affiliations:** a Faculty of Medicine and Health, School of Health Sciences, The University of Sydney, Sydney, Australia; b Allied Health Research Support Centre, Nepean Blue Mountains Local Health District, Sydney, Australia; c School of Education, University of Notre Dame Australia, Sydney, Australia; d School of Health Sciences, University of Notre Dame Australia, Sydney, Australia; e Research Centre in Sports, Health and Human Development, University of Beira Interior, Covilhã, Portugal; f Research and Knowledge Translation Directorate, Mid North Coast Local Health District, C/o Port Macquarie, Australia; g Sydney Catholic Schools, Patrician Brothers College Fairfield, Sydney, Australia; h Institute of Musculoskeletal Health, Sydney Local Health District and The University of Sydney, Sydney, Australia; i Masters and Doctoral Program in Physiotherapy, University of Sao Paulo City, Sao Paulo, Brazil

**Keywords:** Pain education, school, adolescent, health education, pediatric pain

## Abstract

**Background:**

Pain is a significant issue affecting adolescents. However, adolescents often receive outdated, non-evidence-based management for pain. School-based pain education has been identified as a priority for pain management in Australia, yet none is endorsed in schools. This paper describes the process of developing and evaluating the Pain Smart education module for students aged 12–16 at Australian secondary schools.

**Methods:**

The process included five phases: (1) listening to adolescent voices; (2) developing the Pain Smart lessons and resources; (3) piloting the Pain Smart education module; (4) refining the Pain Smart education module; and (5) evaluating the Pain Smart education module. Each phase is mapped to an implementation framework: Phase 1 is aligned with the Consolidated Framework for Implementation Research, Phase 2 and 4 with the Knowledge-to-Action Framework, and Phase 3 and 5 with the Conceptual Framework for Implementation Outcomes.

**Results:**

Phase 1 identified gaps in students’ knowledge and preferences for learning about pain at school. Phase 2 used these findings and evidence-based concepts to develop three curriculum-aligned, ready-to-teach lessons. Phase 3 piloted the module in two secondary schools, demonstrating acceptability, appropriateness and feasibility while identifying issues with study processes. Phase 4 refined the lessons and study processes based on these findings. Phase 5 describes a cluster randomized controlled trial to evaluate the module.

**Conclusions:**

The Pain Smart education module was developed using a comprehensive and collaborative learning approach. Using established implementation frameworks ensured the module addresses real-world barriers and perspectives of relevant parties. Ongoing evaluation will determine its effectiveness and inform its future scalability.

## RÉSUMÉ

**Contexte :** La douleur est un problème important qui touche les adolescents. Pourtant, la prise en charge de la douleur qui leur est proposée repose souvent sur des approches dépassées et non fondées sur des données probantes. L’éducation à la douleur en milieu scolaire a été désignée comme une priorité pour la prise en charge de la douleur en Australie, mais aucun programme de ce type n’a encore été approuvé pour les écoles. Cet article décrit le processus d’élaboration et d’évaluation du module éducatif Pain Smart destiné aux élèves de 12 à 16 ans des écoles secondaires australiennes.

**Méthodes :** Le processus comprenait cinq phases : (1) écouter les voix des adolescents; (2) élaborer les leçons et les ressources Pain Smart; (3) mettre à l’essai le module éducatif Pain Smart; (4) perfectionner le module éducatif Pain Smart; et (5) évaluer le module éducatif Pain Smart. Chaque phase est associée à un cadre de mise en œuvre : la phase 1 au Cadre consolidé pour la recherche sur la mise en œuvre, les phases 2 et 4 au Cadre du passage des connaissances à l’action, et les phases 3 et 5 au Cadre conceptuel des résultats de la mise en œuvre.

**Résultats :** La phase 1 a permis de repérer les lacunes dans les connaissances des élèves et leurs préférences quant à l’apprentissage de la douleur à l’école. La phase 2 s’est appuyée sur ces constatations et sur des concepts fondés sur des données probantes pour élaborer trois leçons conformes aux programmes scolaires et prêtes à être enseignées. La phase 3 a consisté à mettre le module à l’essai dans deux écoles secondaires; cet essai a montré que le module était acceptable, approprié et réalisable, tout en révélant des problèmes liés aux procédures de l’étude. La phase 4 a permis de perfectionner les leçons et les procédures de l’étude à partir de ces constatations. La phase 5 décrit un essai contrôlé randomisé par grappes visant à évaluer le module.

**Conclusions :** Le module éducatif Pain Smart a été élaboré selon une approche d’apprentissage globale et collaborative. Le recours à des cadres de mise en œuvre établis a permis de tenir compte, dans le module, des obstacles rencontrés dans la pratique et des points de vue des parties concernées. L’évaluation continue permettra de déterminer son efficacité et d’orienter son éventuel déploiement à plus grande échelle.

## Introduction

Nearly three-quarters of children and adolescents experience pain at least monthly,^1^ and around 20% experience chronic pain.^2^ Pain can affect all aspects of a young person’s life. Adolescents experiencing pain are more likely to be obese,^3^ have poor mental health,^4,5^ engage in substance use^4,5^ and miss school and sport.^6–8^ They are also more likely to experience chronic pain in adulthood.^9–13^

Pain can impact adolescents’ school functioning and sense of belonging. A large school-based survey (*n* = 48,254, age range 6–17) identified that children with chronic pain have higher odds of chronic absenteeism (OR 4.2 [95% CI 3.0–5.8]), a learning disability (OR 1.6 [95% CI 1.1–2.5]), school-reported problems (1.9 [95% CI 1.5–2.3], and poor school engagement (OR 1.4 [95% CI 1.0–1.9]).^8^ Students with chronic pain also experience social issues at school. Students with chronic pain are more likely to experience bullying than their peers.^14^ In qualitative studies, students with chronic pain report feeling misunderstood and stigmatized by their peers and school personnel, leading to feelings of isolation and withdrawal.^7,15^ Students attribute this to a lack of knowledge and understanding in the school community.^15^

Pain is understood as a biopsychosocial experience, meaning it affects people and is affected by the dynamic interaction of biological, psychological and social factors.^16^ Although this model has been accepted for over 40 years, non-evidence-based information and management for pain consistent with the outdated ‘biomedical model’ remain widespread.^17^ In 2019, the Australian Government commissioned a *National Strategic Action Plan for Pain Management* to address the growing burden of pain in Australia.^18^ The Government subsequently endorsed the *Strategic Action Plan*, which identifies provision of evidence-based information about pain and pain management to the community as a priority, including a call to develop a pain education program for schools. At present, pain education is not included in school health education curricula.

Including pain education within the school curriculum has several potential benefits. Firstly, adolescents often receive non-evidence-based information and management for pain,^19^ and misconceptions about pain are common in healthcare and in the community.^20,21^ School-based pain education ensures that all adolescents have access to evidence-based information about pain and how to manage it. Secondly, experiencing pain during adolescence is associated with experiencing chronic pain during adulthood.^9–13^ Providing adolescents with evidence-based information about pain and pain management at schools could possibly change the trajectory of chronic pain in adulthood. Thirdly, adolescents with chronic pain often experience stigma from peers and school personnel,^7,15^ and have impaired school functioning.^8^ Increasing knowledge and understanding of pain may reduce students’ experiences of stigma and isolation and support greater engagement at school.

Research on comprehensive school-based pain education programs is limited, particularly those embedded with relevant health curriculum. To date, the evidence base on pain education programs delivered to secondary school students includes five short animated videos about pain processing, prevalence and impact of pain in young people and pain management,^22^ two lessons focusing on pain science concepts and role-playing scenarios,^23^ a 10-minute education video about pain neurobiology and pain management,^24,25^ a 30-minute PowerPoint lecture from researchers focusing on Pain Neuroscience Education,^26–29^ and a one-day program delivered by health professionals, including a 70-minute lecture on Pain Science Education, a lived experience talk and group activities.^30,31^ An Australian-based team also piloted a two-part education program in two schools (ages 8–14 years): (i) researcher-led professional development day for teachers about pain science education, and (ii) teacher-led student pain science education program delivered according to teacher preferences.^32^ Few of these interventions were integrated into the curriculum, and only the latter sought input from educators or students and considered their preferences during development.

Implementation science aims to understand how and why implementation of interventions succeed or fail and the magnitude of effect on measured outcomes.^33^ Nilsen describes five categories of theories, models and frameworks in implementation science; *process models* describe and/or guide the process of translating research into practice, *determinant frameworks* describe the barriers and enablers influencing implementation outcomes, *classic theories* are those derived from other fields that can be applied to understand and/or explain aspects of implementation, *implementation theories* are those developed within the implementation field that can be applied to understand and/or explain aspects of implementation, and *evaluation frameworks* provide guidance for evaluating implementation.^33^ A core principle across these frameworks is meaningful engagement with end-users and other relevant and interested parties to reduce research waste and improve the acceptability, appropriateness and feasibility of research methods and interventions.^34–37^

This paper describes the process of developing and evaluating the Pain Smart education module within applicable implementation frameworks. The Pain Smart education module aims to provide adolescents with accurate knowledge and skills to make evidence-informed decisions about managing pain consistent with the biopsychosocial model. This module was developed for secondary school students (ages 12–16) and aligns with the New South Wales (NSW) Government’s Education Authority, the state-level body responsible for education curriculum in NSW, Australia. Given that there is substantial overlap in learning intentions, content descriptors and broad capabilities between the state and national health education curricula, the Pain Smart education module also aligns with the latter.

Pain Smart is part of a broader program of research that aims to increase community awareness of pain and improve outcomes for adolescents with pain. The focus of this current program is to develop and evaluate an evidence-based pain education module embedded in the curriculum, with the aim of aligning students’ understanding of pain with the biopsychosocial model. Accordingly, the primary outcome for the Pain Smart education module presented in this paper is students’ pain knowledge, assessed using the Pain Concepts Questionnaire.^38^

## Methods

### Phases of developing and evaluating the pain smart education module

This paper describes developing and evaluating the Pain Smart education module in five phases. Each phase is aligned with a process, determinant or evaluation implementation framework. We refer to three implementation frameworks in the five phases to capture the conceptual and methodological differences. We used the Consolidated Framework for Implementation Research^39^ (determinant framework) in Phase 1 to understand characteristics of the target population, the Knowledge-to-Action Framework^40^ (process framework) in Phases 2 and 4 to guide the process of developing and refining the intervention, and the Conceptual Framework for Implementation Outcomes^41,42^ (evaluation framework) to provide a structure for evaluating the intervention in Phases 3 and 5.

*Phase 1: Listening to adolescent voices* explores ‘characteristics of the individual’ (Consolidated Framework for Implementation Research) to understand adolescents’ knowledge of pain, attitudes toward school-based pain education, and learning preferences. These insights informed *Phase 2: Developing the Pain Smart lessons and resources*, a collaborative ‘knowledge creation’ process (Knowledge-to-Action Framework) that led to the development of three curriculum-aligned, ready-to-teach lessons. These lessons were piloted in *Phase 3: Piloting the Pain Smart education module*, and the implementation outcomes related to the individual provider (teacher), individual consumer (student) and organizational level (principal) (Conceptual Framework for Implementation Outcomes) were evaluated. The findings from the pilot informed *Phase 4: Refining the Pain Smart education module*, in which the intervention and study processes were adapted for classroom delivery (Knowledge-to-Action Framework). *Phase 5: Evaluating the Pain Smart education module* describes a cluster randomized controlled trial to evaluate implementation and service outcomes (Conceptual Framework for Implementation Outcomes) of the Pain Smart education module.

The structure of each phase is organized around guiding questions outlined at the start of each section.

### Phase 1: listening to adolescent voices

Three questions guided this development phase:

1. How do adolescents conceptualize pain?
2. What do adolescents want to learn about pain at school?
3. How do adolescents want to learn about pain at school?

The Consolidated Framework for Implementation Research (CFIR) is a determinant framework that directs systematic assessment of barriers and enablers to the implementation of an intervention in real-world settings across five domains.^39^ One domain (“characteristics of the individual”) examines how a person’s attitudes toward the intervention, their perceived value of it, their familiarity with the facts and principles related to the intervention, and their motivation to engage can influence its implementation.

To ensure the final module was relevant to the adolescents who would receive it, we conducted interviews with 25 adolescents^43^ and surveyed 501 adolescents.^44^ These two studies aimed to identify (1) gaps in adolescents’ knowledge about pain, (2) topics about pain they value, and (3) their preferences for engaging with pain education in the classroom. This ensured that the Pain Smart education module reflected adolescents’ knowledge, attitudes and motivations regarding pain and school-based pain education. In both studies, adolescent coauthors provided input on study methods and interpretation of findings. Ethics approval for both studies was obtained from the University of Sydney Human Research Ethics Committee (HREC approval numbers: 2023/403 and 2023/592).

#### How do adolescents conceptualize pain?

Participants in the qualitative study conceptualized physical and psychological pain as distinct experiences: physical pain mostly related to injuries, and psychological pain captured experiences such as grief and depression. While participants understood psychological and social factors influence and are influenced by pain, they tended to rely on biomedical treatments such as first aid and medication to manage pain. Participants expected pain to resolve within a short timeframe, and pain persisting beyond this timeframe indicated serious injury, delayed healing or requiring further investigation.

Participants held some beliefs about pain that could result in stigma related to chronic pain. There were conflicting views about whether people experience pain differently, or whether people experience pain similarly but react differently. The latter was underpinned by beliefs that some people tolerate pain better and are more resilient than others. Participants expected pain to be short-term and visible, and those who experienced pain without visible signs of injury or who had persistent pain reported feeling misunderstood by their family and peers.

#### What do adolescents want to learn about pain?

In the survey of 501 adolescents, 70% agreed or strongly agreed that it is important to learn about pain. The top three topics adolescents wanted to learn about pain were: ways to treat pain (71%), different types of pain (67%) and the role of the nervous system (62%).

#### How do adolescents want to learn about pain at school?

Of the 501 adolescents surveyed, 84% preferred to learn about their pain from health professionals, followed by teachers (48%). Nearly half of the adolescents (49%) preferred learning about pain through a combination of in-person and online modalities, with the highest preference for “watching an educational video and having a class discussion.” There was a stronger preference for interactive approaches such as class discussions, class presentations and group activities, compared to less interactive approaches such as worksheets, animations or recorded presentations.

#### Phase 1 summary

This phase describes how we considered characteristics of the population receiving the Pain Smart education module. The CFIR suggests that understanding the target population’s attitudes toward the intervention, their perceived value, their familiarity with the relevant principles, and their motivation to engage with the intervention can influence implementation. These studies highlight that adolescents were motivated to engage in pain education in school, could identify topics they value, and offered insight into ways to engage them in learning. By exploring their familiarity with pain and preferred ways of learning, we identified clear learning objectives for pain education that informed Phase 2.

#### Phase 1 reflection

Exploring teacher characteristics, such as their understanding of pain and attitudes toward school-based pain education could have provided greater insight into implementation determinants. Teachers’ own experiences of pain often differ from their understanding of students’ pain^45^ and they report difficulty supporting students with chronic pain.^46^ As such, teachers’ conceptualization and beliefs about pain may influence how they engage with and deliver the intervention. Including teachers’ perspectives could have provided greater insight into content development, teacher training needs and enablers for teacher engagement with the lessons. Further, a more comprehensive assessment of the inner setting factors, such as school social architecture, school values and culture, and implementation climate could have provided valuable insights into other determinant factors that could affect delivery of the intervention.^39^

### Phase 2: developing the pain smart lessons and resources

This development phase was guided by two questions:

1. What content should be included in the Pain Smart lessons?
2. How do we convert the content into a format that is deliverable in the classroom?

The Knowledge-to-Action Framework is a process framework that describes the process of translating research into practice-bridging ‘knowledge creation’ with ‘knowledge application.’^40^ Knowledge creation describes generating knowledge through knowledge inquiry (primary research), knowledge synthesis (literature review) and knowledge tools/products (guidelines and tools), and the action cycle describes the process of implementing the knowledge in real-world settings.

The process of developing the Pain Smart education module included two parts: identifying content to be included in the lessons (knowledge creation) and delivering the Pain Smart education module in the classroom (knowledge application). This phase describes ‘knowledge creation.’

#### What content should be included in the pain smart lessons?

After understanding how adolescents conceptualize pain through the survey and interviews, we identified where their knowledge was consistent with and where it contradicted contemporary biopsychosocial understanding of pain. We also reviewed literature to identify key evidence-based concepts and considered the topics valued by the adolescents. See Table 1 for a summary of key findings, evidence-based concepts, possible gaps in knowledge and learning objectives. We developed the content for three lessons about pain and pain management reflecting their knowledge gaps, preferences and current scientific understanding of pain.

**Table 1.** Learning outcomes for pain education identified from study findings.

Study findings	Evidence-based concepts	Gaps in knowledge	Learning outcomes
“Physical” pain is mostly due to injury.	Pain may or may not be related to tissue damage (injury).^16^	Narrow definition of pain, where pain is analogous to injury.	**Define** pain
Physical and psychological (“mental” or “emotional”) pain are distinct and processed differently by the body.	Pain is a sensory and emotional experience. Information about danger is sent from the peripheral to central nervous system. Depending on how the CNS process this information, pain may be experienced.^16^	Incomplete understanding about pain processing.	**Explain** the role of the central and peripheral nervous systems.
Persistent pain indicates serious injury, delayed healing or needing further tests.	Injuries typically heal within a defined time frame, but pain can persist beyond the time it takes for injury to heal.^16^ This can be due to ongoing disease processes or changes in the peripheral and/or central nervous systems.^47^	Insufficient explanation for persistent pain	**Differentiate** between acute and chronic pain
Psychological and social factors influence how pain is experienced, but some people can ‘tolerate’ pain better than others.	Pain is an individual and subjective experience that influences and is influenced by biological, psychological and social factors.^47^	Incomplete understanding of biopsychosocial experience of pain	**Develop** understanding of the biopsychosocial experience of pain
Physical pain is mostly managed using biomedical strategies (e.g., first aid, medication, exercise).	Psychological and social strategies are useful in managing pain.^48^	Limited use or knowledge of psychosocial strategies (e.g., relaxation, social support, psychological therapies)	**Explore** biopsychosocial management strategies
People who can ‘tolerate’ or ‘endure’ pain are resilient.	Pain is a subjective experience, meaning people feel and react to pain differently.^16,47^	Possible stigmatizing beliefs about how individuals experience pain	**Discuss** how stigma can affect people experiencing pain

Table adapted from Bogard I, Ayre J, Smith J Exploring Adolescents’ Understanding, Experiences and Beliefs About Pain: A Qualitative Study. Health expect 2024;27(6):e70132. https://doi.org/10.1111/hex.70132Note: The words in **bold** indicate the level of metacognition to be demonstrated.

#### How do we convert this content into a format that is deliverable in the classroom?

Although surveyed adolescents preferred to learn about pain from health professionals, we chose to have the lessons delivered by health education teachers (their second preference) to enhance the feasibility of delivering the module in the classroom and support its long-term adoption and scalability.

We collaborated with an experienced health education teacher and curriculum writer, who later became a member of the research team, to convert the content into lessons and align the module with the health education curriculum. This iterative process occurred over a 10-month period with multiple feedback rounds. The output was three ready-to-teach lessons aligned with stages 4 and 5 (ages 12–16) of the PDHPE curriculum.

The lessons were developed in a format that reflects current teaching practices and students’ preferences for learning about pain at school. The Pain Smart module included three PowerPoint presentations (one for each lesson), a teacher handbook and a student workbook. In developing the lessons, we used concepts from the Interactive-Constructive-Active-Passive (ICAP) framework, which describes how different modes of learning lead to different knowledge-change processes.^49^ For example, the interactive mode of engagement describes constructive dialogue between at least two people, which can lead to greater change in knowledge through the process of co-inferring.^49^ We used a range of learning activities in the lessons to correspond with different modes of learning:

- Student-centered and interactive learning approaches, including constructivist tasks, guided class discussions and socio-cultural learning activities where students collaborated to build shared understandings of pain. For example, students watched an educational video about pain processing in the nervous system and participated in a guided discussion about key concepts and reflections on how this may have changed their understanding of pain.
- Explicit and inquiry-oriented instruction, combining direct teaching of core concepts with inquiry-based questioning and case-based problem-solving activities that enabled students to apply the biopsychosocial model to authentic scenarios. For example, after learning about the biopsychosocial model, students identified the relevant biopsychosocial factors in various case scenarios, brainstormed possible biopsychosocial management strategies, and reflected on possible barriers and solutions to implementing these strategies.
- Reflective and health-literacy development activities, such as personal reflection prompts and analysis of misconceptions to strengthen students’ critical thinking, self-awareness, and capacity to make informed health decisions. For example, students reflected on how stigma can affect young people experiencing chronic pain and discussed ways to address pain-related stigma.

We aligned the module with stages 4 and 5 of the NSW Personal Development Health and Physical Education (PDHPE) curricula. We translated the identified knowledge gaps into learning objectives using language consistent with the curriculum, and embedded it within the learning strand ‘Safe, active and healthy lifestyles.’ Table 1 summarizes how the learning objectives map to the qualitative and survey study findings, evidence-based concepts and gaps in knowledge. The identified learning outcomes also align with adolescents’ preferences for learning about pain identified in the survey.

The learning outcomes were distributed across three lessons:

1. Lesson One: understanding pain and its impact
  - Define pain
  - Explain the role of the peripheral and central nervous system
  - Differentiate between acute and chronic pain
2. Lesson Two: impact of pain on the whole person
  - Develop understanding of the biopsychosocial model of pain
3. Lesson Three: smart and healthy ways to manage pain
  - Explore biopsychosocial management strategies for pain
  - Discuss how stigma can affect people experiencing pain

To support module delivery, we developed a 1-hour teacher training session outlining the rationale for the Pain Smart lessons, their development, key content and concepts, and the delivery approach. Sessions were delivered in small, interactive groups, either in-person or online. We also developed a two-page summary of the lessons for caregivers.

#### Phase 2 summary

This phase describes the process of knowledge creation aligned with the Knowledge-to-Action Framework.^40^ We integrated the knowledge we generated from the survey and interview studies (knowledge inquiry) and current evidence on pain (knowledge synthesis) to develop content for three lessons. Collaborating with educators, we then adapted the lessons into a format aligned with contemporary teaching practices and suitable for the PDHPE classroom. The resultant Pain Smart education module was piloted in Phase 3.

#### Phase 2 reflection

The KTA framework does not explicitly describe involving end-users during knowledge creation, however it is widely accepted that including end-users in the development of health interventions can improve research quality and acceptability of interventions.^35,37^ We included adolescent perspectives in Phase 1; but a more collaborative partnership could have provided greater insight into language, design and activity preferences. Further, including more teachers in this phase could have provided more diverse perspectives and enhanced applicability.

### Phase 3: piloting the pain smart education module

This evaluation phase was guided by two questions:

1. How do teachers and students perceive the Pain Smart lessons?
2. How feasible are the Pain Smart study processes in terms of consent rates and data completeness?

The Conceptual Framework for Implementation Outcomes is an evaluation framework that distinguishes between three outcome types: implementation outcomes, service outcomes and client outcomes.^41,42^ Implementation outcomes may relate to the individual provider or consumer of an intervention (e.g., acceptability, adoption, appropriateness, feasibility and fidelity), the organization or setting in which it is delivered (e.g., penetration and sustainability) and the provider or providing institution of the intervention (e.g., cost).^42^

The Pain Smart education module was piloted in two secondary schools, delivered by 9 PDHPE teachers to 312 students in grades 8–10 (Under review, Bogard, 2026). The pilot aimed to evaluate implementation outcomes at the individual provider (teacher), individual consumer (student) and organizational level (principal). The feasibility trial was prospectively registered on the Australia New Zealand Clinical Trials Registry (registration number: ACTRN12624000253505), the protocol was publicly registered on the Open Science Framework (https://osf.io/wb9ng/) and ethics approval was obtained from the University of Sydney (approval number 2023/HE000781).

#### How do teachers and students perceive the pain smart lessons?

We asked students to complete a feedback survey after each of the three lessons and at the end of the study (3-months). Teachers completed feedback surveys after each lesson and were invited to semi-structured interviews following the final lesson to provide provider-level insights. Principals were invited to participate in a semi-structured interview after the study to evaluate from an organizational perspective. Table 2 describes how implementation outcomes were evaluated in the pilot.

**Table 2.** Evaluation of implementation outcomes in the Pain Smart pilot.

Level of analysis	Implementation outcome	Measure
**Individual consumer: *Students***	**Acceptability** of the lessons	Theoretical Framework of Acceptability^50^ Affective attitude (‘engaging’) Perceived effectiveness (‘useful information about pain’) Intervention coherence (‘relevant to me and my peers’ and ‘easy to understand’) General acceptability (open ended question)
	**Acceptability** of the study	Theoretical Framework of Acceptability^50^ Burden (‘participating took a lot of effort’) Intervention coherence (‘important for teenagers to learn about pain’) Perceived effectiveness (‘greater understanding of pain’) General acceptability (open ended question)
**Individual provider**: ***Teachers***	**Acceptability**	Acceptability of Intervention Measure^51^ Meets approval Is appealing Likeable Welcome Semi-structured interviews ‘Overall, what was your experience teaching the Pain Smart lessons to your students?’ ‘In your opinion, how well did the students engage with the content? Did you get any feedback from the students?’
	**Appropriateness**	Intervention Appropriateness Measure^51^ Fitting with the PDHPE syllabus Suitable for students in grades 7–10 Applicable to students in grades 7–10 Good match with the current PDHPE syllabus Semi-structured interviews: ‘In your opinion, how well does this content fit with the current PDHPE syllabus?’ ‘In your opinion, do you think it’s important for teenagers to learn about pain at school?’
	**Feasibility**	Feasibility of Intervention Measure^51^ Implementable in the classroom Possible to implement on a larger scale Doable Easy to use Semi-structured interviews ‘Which elements of the lessons worked well in the classroom, and which elements didn’t?’
	**Fidelity**	Semi-structured interviews ‘Did you make any modifications to the content, if so, what changes did you make?’
**Organization**: ***Principal***	**Adoption**	Semi-structured interview ‘What factors motivated you to engage with the study?’ ‘Did you have to overcome any barriers or resistance when choosing to participate, if so, what were they?’ ‘In your opinion, what would make it easy for other schools to engage in this study?’
	**Sustainability**	Semi-structured interview ‘Do you see any barriers to implementing this education module into schools long-term, if so, what are they?’ ‘What would make it easier to integrate this education module into schools long term?’

The students who completed the feedback surveys rated the three lessons as engaging, relevant, useful and easy to understand, reflecting high acceptability. However, low response rates warrant cautious interpretation of these findings. Teachers rated all lessons as acceptable, appropriate for grade 7–10 PDHPE students and feasible to implement in the classroom. Teachers reported disruptions to the school calendar, and student absence affected the timing of lesson delivery and completion of lesson content, affecting intervention fidelity. The principal and PDHPE coordinator providing feedback at the organizational level reported that the Pain Smart education module was well resourced, reduced lesson preparation time and fit within the existing curriculum. They highlighted teacher time and competing priorities as possible barriers to implementing the education module into the curriculum long term, especially as the new PDHPE curriculum is introduced in 2027. The full results can be found in the published paper (Under review, Bogard, 2026).

#### How feasible are the pain smart study processes in terms of consent rates and data completeness?

Difficulties with student consent, data completeness, teacher absence and competing priorities at the pilot schools affected our ability to evaluate implementation outcomes as intended. All 312 students participated in the lessons; however, we required opt-in parental consent and student assent to participate in data collection. Of the 312 eligible students, only 26 (8%) had both parental and student consent for data collection. Students required parental consent to receive the baseline survey and needed to complete the baseline survey to receive subsequent surveys. Despite allocating time within the lessons to complete the surveys, only 34% of students who received the baseline survey completed it, and only 23% of those students completed the 3-month follow-up survey. Between 4–10 students completed feedback surveys for each lesson, and 8 teachers provided feedback for lessons one and two, and 5 provided feedback for lesson 3. Student and staff absence and issues accessing the survey link affected survey completion.

#### Phase 3 summary

This phase evaluated the Pain Smart education module and study processes aligned with the Conceptual Framework for Implementation Outcomes.^41,42^ The implementation outcomes evaluated reflect high consumer acceptability, high provider acceptability, appropriateness and feasibility, and highlighted possible barriers and enablers to adoption and sustainability at the organizational level. The most serious issues were very low consent and student data completeness, which reflect problems with study operational processes, including an over-reliance on school-generated e-mails to obtain parental and student consent. These findings were incorporated into Phase 4 to refine the intervention and study processes.

#### Phase 3 reflection

Low consent and data completion rates limited our evaluation of provider fidelity, consumer acceptability and organizational adoption and sustainability. Although we included one interview question to assess teacher fidelity, the low response rates to teacher interviews meant that fidelity could not be adequately assessed. Including additional measures such as a teacher checklist of core lesson components, or researcher observation of the lessons could have strengthened fidelity evaluation. We surveyed students about the acceptability of the lesson and anticipated approximately 300 student responses. However, the low data completion rate limited our ability to adequately assess this outcome. Embedding a qualitative component could have strengthened student acceptability assessment. We were only able to interview one principal, meaning assessment of barriers and facilitators to organizational adoption and sustainability was limited.

### Phase 4: refining the pain smart education module

This refinement phase was guided by two questions:

1. How can we improve the Pain Smart education module based on pilot findings?
2. How can we improve the Pain Smart study processes based on pilot findings?

As described in Phase Two, the Knowledge-to-Action Framework is a process framework that describes the process of bridging ‘knowledge creation’ with ‘knowledge application.’^40^ Phase 2 of the study described the ‘knowledge creation’ phase. ‘Knowledge application’ involves adapting the knowledge to the local context, assessing barriers to using the knowledge, tailoring and implementing the interventions, monitoring knowledge use, evaluating outcomes and sustaining knowledge use.^40^

This phase reflects the ‘knowledge application’ stage, describing how the Pain Smart education module and study processes were adapted and tailored following the pilot study at two schools.

#### How can we improve the pain smart education module based on pilot findings?

Feedback from teachers and the principal informed revisions to the Pain Smart education module, as outlined in Table 3.

**Table 3.** Refining of the Pain Smart education module based on pilot findings.

Teacher feedback from pilot	Change to the Pain Smart education module
Some students struggled to understand the term ‘biopsychosocial’ in lesson two.	We allocated more time to understanding each individual component of the term ‘bio-psycho-social.’
Younger grades may struggle with the amount of content in the module.	We developed a four-lesson version that delivers lesson two in two parts. Teachers can choose which is more appropriate for their students.
Older grades or advanced classes may benefit from additional activities that focus on applying their knowledge.	We developed an additional research activity where students identify a well-known person (e.g., sportsperson, celebrity) experiencing pain and apply the biopsychosocial model of pain and pain management to their situation.
Aligning the lessons with the new PDHPE syllabus would lead to greater uptake by schools.	We aligned the Pain Smart education module with stages 4 and 5 of the new PDHPE syllabus, due for implementation in 2027.

In addition to these changes, we consulted the Pain Smart youth advisory group to develop the Lesson 3 content on stigma. The youth advisory group includes 18 Australian adolescents aged 12–18 years, with and without lived experience of pain. This process resulted in changes to the definition and explanation of stigma, including why adolescents with chronic pain may experience it, and the inclusion of a case study to prompt reflection and discussion about how stigma may be experienced by adolescents with pain.

#### How can we improve the pain smart study processes based on pilot findings?

Findings from the pilot study, including feedback from teachers and principals, offered valuable insights to refine the Pain Smart study processes for future research. To improve consent rates, we will offer both opt-in and opt-out consent methods, and electronic and paper consent forms for schools to choose. Schools will be recompensed for their involvement in the study, and students will be eligible to win a gift card for participating. To improve data completeness, members of the research team will be present in the classroom during data collection.

#### Phase 4 summary

This phase describes the process of Knowledge Application of the Knowledge-to-Action Framework,^40^ where the Pain Smart education module and study processes were adapted following classroom delivery in the pilot. These refinements aligned the lessons with classroom needs, addressed barriers to integration within the PDPHE curriculum, and addressed barriers to effective evaluation of the Pain Smart education module in future research. Engagement with the Pain Smart youth advisory group led to meaningful improvements in the stigma content. The plans for the planned evaluation of the refined Pain Smart education module are described in Phase 5.

#### Phase 4 reflection

Greater collaboration with end-users during Phase 4 could have enhanced classroom applicability. Although we incorporated the pilot findings, we did not reengage with teachers to ensure these refinements reflected their feedback. In addition, the low student response rate to acceptability surveys limited the inclusion of their perspectives in Phase 3. More active engagement with adolescents during this phase may have helped ensure their views were better represented in lesson refinements.

### Phase 5: evaluating the pain smart education module

This evaluation phase is guided by two questions:

1. How effective is the Pain Smart education module in improving students’ understanding of pain?
2. How can we encourage schools to participate in the evaluation of the Pain Smart education module?

As described in phase 3, the Conceptual Framework for Implementation Outcomes is an evaluation framework that describes implementation outcomes, service outcomes and client outcomes.^41,42^ Implementation outcomes relate to the individual provider, consumer and organization, and service outcomes relate to the quality of the intervention (e.g., effectiveness and efficiency).^42^

This phase describes the evaluation of effectiveness of the Pain Smart education module (service outcomes), with embedded process evaluation (implementation outcomes).

#### How effective is the pain smart education module in improving students’ understanding of pain?

We are conducting a cluster randomized controlled trial of secondary schools to evaluate the effectiveness of the Pain Smart education module in improving students’ understanding of pain. All mainstream schools in NSW, Australia are eligible to participate, and no students attending participating schools are excluded. Students with learning or language difficulties are supported by the school’s diverse learning, and/or English as an Additional Language or Dialect teams.

To measure the effectiveness of the education module, schools are randomly allocated (1:1 ratio) to receive the Pain Smart education module immediately, or after the 12-month data collection phase. A cluster-randomized design with the school as the unit of randomization is used to minimize cross-group contamination. The primary outcome measure is the mean difference between groups in pain understanding at 3-months, measured using the Pain Concepts Questionnaire.^38^ Secondary outcomes include mean difference between groups in pain interference for students reporting problems with pain, depression and anxiety, school absence due to pain, and smoking and alcohol use. All outcome measures are reassessed at 12 months to determine whether the intervention effects are maintained over time.

The trial was prospectively registered on the Australia New Zealand Clinical Trials Registry (registration number: ACTRN12625000793415), the protocol has been published (Under review, Bogard, 2026), and ethics approval has been obtained from the University of Sydney (project identifier 2024-HE001722).

#### How can we encourage schools to participate in the evaluation of the pain smart education module?

We will implement various strategies to encourage schools to participate in the Pain Smart trial. These strategies have been informed by previous school-based health intervention research,^52–55^ implementation frameworks,^39,56^ and insights from our collaborators working within the school sector. To improve knowledge of the study, we will use social media advertising to reach potential parent and teacher champions and engage with educator networks and school associations. To increase educator buy-in, we have aligned the intervention with the health education curriculum, we will identify and support school champions and will provide school and student financial incentives to participate. To ensure teachers are supported, we will provide teachers with a comprehensive evidence-based education module with all teaching resources included and teacher training and ongoing support throughout the study. To demonstrate institutional fit and sustainability, we have aligned the intervention with the education department’s strategic plan to improve student wellbeing and learning and will provide schools with school-level feedback to demonstrate the value of the Pain Smart education module on students’ knowledge and wellbeing. These strategies aim to increase participation in the Pain Smart clinical trial to ensure thorough evaluation of the Pain Smart education module.

We will embed a process evaluation within the Pain Smart clinical trial. We will evaluate provider level (teacher) acceptability, appropriateness, feasibility and any lesson adaptations using the same instruments as the pilot study described in Phase 3,^51^ and two additional open-ended questions about any adaptations to the lessons they made when delivering the module or would suggest for future iterations.

#### Phase 5 summary

This phase describes plans to evaluate elements of the implementation and service outcomes identified in the Conceptual Framework for Implementation Outcomes.^41,42^ The cluster RCT will evaluate the effectiveness of the Pain Smart education module in improving adolescents’ understanding of pain with embedded evaluation of the provider-rated acceptability, appropriateness, feasibility and fidelity of the intervention.

## Discussion

The Pain Smart education module was developed through a multi-phase approach, underpinned by process, determinant and evaluation frameworks. Conduct of Phase 1–4 and planning of Phase 5 have been completed over a period of 3 years. Using established implementation frameworks ensures the Pain Smart education module addresses real-world barriers, contextual factors and considers the perspectives of various relevant and interested parties, including adolescents, educators, and researchers. This approach enhances contextual fit, ensures applicability of the Pain Smart education module to the classroom and facilitates its future scalability.

The first four phases produced clear outcomes that directly informed the phases that followed. Phase 1 engaged adolescents through primary research and coauthorship to inform content that was appropriate for young people and aligned with their learning values. Phase 2 translated primary research and scientific knowledge into ready-to-teach, syllabus-aligned lessons informed by pedagogical approaches. Phase 3 evaluated the feasibility of the Pain Smart study and identified refinements to both the education module and study procedures to support future evaluation. Phase 4 adapted the intervention in response to educator and student feedback to enhance its relevance and acceptability, and refined study processes to align with school contexts and enable large-scale evaluation. Phase 5 describes the planned evaluation of the Pain Smart education module.

As the Pain Smart education module is delivered in an education context, the extent to which health-based implementation frameworks aligned varied. Process frameworks describe and guide the process of translating research into practice through a series of steps.^33^ As evidenced in the Knowledge-to-Action framework used in Phases 2 and 4, the steps are general in nature, and hence easily applicable across health and education settings.^40^ Determinant frameworks describe a ‘systems’ approach and aim to understand and explain what influences implementation at multiple levels of influence.^33^ While the determinants are different across school and health settings, both are complex systems that require consideration of the dynamic relationship between and within the interventio and inner settings, and characteristics of individuals, for successful implementation.^39^ Given that the education and health sectors are driven by different needs, evaluation frameworks may be more context specific. For example, we only aligned Phase 3 with the implementation outcomes of the Conceptual Framework for Implementation Outcomes, and Phase 5 with effectiveness in the service outcomes, as the other service outcomes related to safety, efficiency, patient-centeredness and timeliness, and client outcomes related to symptomology and function, which were not relevant in the education setting.^42^

There are differences between education and health that should be considered when applying implementation frameworks. While these contexts share commonalities, such as the need for strong leadership, access to ongoing resources and training, and intervention adaptability, a key difference is that schools prioritize educational outcomes over health outcomes.^57^ The School Implementation Strategies, Translating ERIC Resources (SISTER) strategy compilation refined the Expert Recommendations for Implementing Change (ERIC) strategy^58^ for use in school contexts, resulting in 75 unique school-based implementation strategies.^59^ Most of the adaptations were superficial (i.e., changing terminology, referents or examples), some strategies were deleted, and substantial changes were made to evaluative strategies by shifting focus from patient outcomes and standards to the extent to which school personnel implemented the core components of the intervention.^59^ The SISTER compilation also added seven additional strategies related to providing interactive assistance, adapting and tailoring interventions to context, supporting educators and building change infrastructure. Researchers looking to implement school-based health interventions should consider the unique implementation strategies relevant to schools.

Implementing health interventions in schools can be challenging. Common barriers to implementation and sustainability include staff turnover (e.g., new leadership or loss of a school champion), limited student engagement, teachers not perceiving the benefits for student wellbeing, teachers not feeling confident delivering content outside their expertise, and inflexible interventions.^57^ Partnerships are a key facilitator in the implementation and sustainability of health interventions in schools.^57,60^ Establishing these partnerships is often a combination of deliberate strategy and opportunity. Strategies we used to develop partnerships included engaging with key personnel within local school networks, building existing relationships and developing collaborative research partnerships.

Strengths of the Pain Smart development and evaluation process include a comprehensive, multi-phased approach, developed with input from important relevant and interested parties including adolescents, educators, researchers and clinicians. This resulted in development of an evidence-based intervention that is relevant to Australian adolescents and is deliverable in the classroom. The limitations are described in the summary section of each phase. Overall, we did not explore teacher characteristics in Phase 1, which limited assessment of possible barriers to intervention delivery and effectiveness of knowledge transfer. We had limited engagement with end-users in Phases 2 and 4, which may impact the acceptability, appropriateness and feasibility of the Pain Smart module. Low consent and data completion rates affected assessment of student and teacher implementation outcomes in Phase 3, which influenced the scope of refinements in Phase 4.

Possible future directions include the wide-scale implementation of Pain Smart across all Australian schools. Drawing on the Consolidated Framework for Implementation Research^39^ and findings from the Pain Smart clinical trial, an implementation strategy can be developed to address key barriers and enablers to implementation. The effectiveness of these strategies can then be evaluated in an implementation trial, using established implementation outcomes such as those identified in the RE-AIM framework.^61^ The current Pain Smart program focuses on curriculum changes to improve students’ knowledge about pain, which The World Health Organization Health Promoting School Framework suggests should be part of a multi-component interventions aimed at better outcomes in adolescent health.^52,62^ Future directions for the Pain Smart program of work could include components targeting school ethos and environment and engagement with families and communities, which may lead to health improvements beyond knowledge.^62^ Further research should also explore teachers’ understanding and beliefs about pain, and any impact this has on delivering pain education in the classroom.

In conclusion, the development and evaluation of the Pain Smart education module has been a comprehensive, collaborative process involving clinicians, researchers, educators and adolescents, aligning with relevant process, determinant and evaluation implementation frameworks. The implementation frameworks have provided a structure for intervention development and evaluation, implementation in a real-world setting, critical reflection on research methods, and avenues for future research.
